# Perceived Institutional Restraint Is Associated With Psychological Distress in Forensic Psychiatric Inpatients

**DOI:** 10.3389/fpsyt.2019.00410

**Published:** 2019-06-11

**Authors:** Irina Franke, Michael Büsselmann, Judith Streb, Manuela Dudeck

**Affiliations:** ^1^Department of Forensic Psychiatry and Psychotherapy, Ulm University, Ulm, Germany; ^2^Psychiatric Services Graubuenden, Department of Forensic Psychiatry, Cazis, Switzerland

**Keywords:** restraint, forensic psychiatry, psychological distress, perceived coercion, mandated treatment, suicidal ideation

## Abstract

**Background:** Patients in forensic mental health care experience internal and external coercion; the latter comprises different levels of institutional restraint. These restrictions of individual freedom are mainly justified by the safety interests of third parties and are not necessarily in the patients’ best interests. The effects of such a setting on mentally disordered offenders’ psychological state and treatment course are not fully understood. Assessing both patients’ perception of restraint and psychopathological symptoms would allow us to better understand how restraint and psychopathology interact and how they might influence treatment.

**Methods:** In 184 forensic psychiatric inpatients, we assessed perception of institutional restraint with an adapted version of the Measuring the Quality of Prison Life (aMQPL) questionnaire and current psychological state with the Brief Symptom Checklist (BSCL) and Beck Hopelessness Scale (BHS).

**Results:** Perceived institutional restraint (as expressed in the aMPQL subscales *Transparency of procedures and decisions*, *Fairness*, and *Respect*) was associated with a higher general level of psychological symptoms. Furthermore, patients who perceived a lack of institutional transparency and respect were more likely to have higher scores for hostility, depression, and suicidal ideation. We also found age and sex differences, with higher levels of psychological symptoms in younger and female patients. The diagnosis and duration of detention did not relate to perceived restraint.

**Discussion:** Our results indicate that certain aspects of institutional restraint in long-term forensic inpatient settings correlate with certain psychological symptoms. The observed association might be explained by different kinds of factors: institutional (custodial focus), individual (self-efficacy, diagnosis, and personality), and situational (duration of detention). Although not all of these explanatory factors were addressed by the present study design, forensic mental health professionals should be aware of the relationship between perceived institutional restriction and psychopathology because it might influence treatment course and outcome.

## Introduction

Although coercion in forensic psychiatry shares common features with coercion in general psychiatry, it differs significantly concerning the justification of these measures on the basis of public safety interests, not only individual treatment goals. In forensic psychiatry, in virtually every case, the admission to treatment itself is a compulsory intervention. This difference has a major impact on the autonomy and freedom of forensic psychiatric inpatients and on the balance of power between staff and inpatients, and the structural and institutional features of forensic psychiatric inpatient settings are often more similar to those of prisons than health care settings. This raises the questions whether and how these conditions might influence the psychological state and treatment of mentally disordered offenders.

In psychiatry, coercion has been conceptualized as an external/objective action or an internal, subjective attitude (perceived coercion) or both and as often resulting from a compulsory action (1). Applied to forensic psychiatry, external coercion can be direct (such as involuntary medication or seclusion) and indirect (such as rules and regulations, decision making, atmosphere, and communication), whereby the latter represents institutional coercion. A review on direct physical coercion (i.e., seclusion, restraint, and involuntary medication) in forensic psychiatry found that younger and newly admitted patients tended to be secluded more often than older patients and that female patients were more likely to be restrained and secluded than male patients; furthermore, female patients tended to be restrained or secluded as a result of self-harm, whereas male patients were secluded or restrained as a result of harming others (2). Compared with general psychiatry [e.g., Refs. (3–5)], studies on the outcome coercive measures in forensic psychiatric treatment are rare.

To date, research on patients’ perspectives of coercion in forensic psychiatry has mainly been performed on external, physical forms of coercion, namely, restraint, seclusion, and involuntary medication. In a comparison of forensic psychiatric patients’ and general psychiatric patients’ view of the experience of seclusion, the former more often described their seclusion as punishment, while one-third of both groups claimed not to understand why they were secluded (6). Another study in forensic psychiatric inpatients showed that patients had a negative perception of coercive treatment; however, over half of them declared that the treatment was necessary (mainly to prevent violence), and 16% to 36% even reported that the last episode of coercive treatment had been a positive experience (7). The most frequently reported negative effects of coercion were fear, loss of dignity, humiliation, and fearful loss of control (7). One can hypothesize that these effects might be greater if physical coercion occurs in an institutional context perceived as being highly restrictive. Furthermore, patients’ perception of institutional coercion might have relevant effects on their psychological state, motivation, insight, and readiness to change.

Research on perceived institutional restraint in forensic psychiatry is rare. A recent review conceptualized perceived restrictiveness in forensic care across individual (i.e., relational, tangible), institutional (i.e., built environment, activities, culture, atmosphere, therapeutic aspects, security, practicality), and systemic (i.e., regulatory, temporal) levels; the amount of perceived restrictiveness depended on whether the focus of care was more caring (vs. custodial) and whether the resident was rated as risky (8). The authors stated that because of the negative outcomes of restrictive measures, it is necessary to reflect critically on practices, procedures, and policies in forensic care settings (8). Therefore, it seems important to better understand whether and how indirect coercion is associated with psychopathology. In this study, we focused on the relationship of institutional coercion with the psychological state of forensic psychiatric inpatients. We expected that patients who perceived their institution’s measures as being overly restrictive, unfair, and arbitrary may have more difficulties with adjustment, expressed by a higher rate and broader range of psychological problems. The results might be relevant for establishing and evaluating institutional cultures in forensic mental health care.

## Methods

### Participants

Forensic psychiatric inpatients were included if they were 18 years or older and if, in the opinion of the professionals responsible for their treatment, they were able to give informed consent (i.e., if they had no acute symptoms of a mental disorder and no intellectual disability). In total, *N* = 184 forensic psychiatric inpatients (female = 25) participated in the study. Because of missing values, the data of 54 participants were excluded. Thus, the final sample comprised 130 patients (female = 20). The patients were recruited between February and August 2018 at 10 of the 14 forensic mental health hospitals in the state of Bavaria, Germany. All patients were detained according to Section 63 (severe mental disorder, *n* = 52; 40%) or Section 64 (substance use disorder, *n* = 78; 60%) of the German penal code. The patients had a mean age of 35.64 years [range, 19–68; standard deviation (SD), 11.34] and had been treated for a mean of 34.18 months (range, 0–344; SD, 56.60). They were diagnosed with the following disorders according to International Statistical Classification of Diseases and Related Health Problems, 10th Revision (ICD-10) criteria: substance-related disorder alone (*n* = 65; 50%), personality disorder alone (*n* = 20; 15%), schizophrenia alone (*n* = 17; 13%), depression alone (*n* = 2; 2%), comorbid substance-related disorder and personality disorder (*n* = 12; 9%), comorbid schizophrenia and personality disorder (*n* = 3; 2%), comorbid substance-related disorder and schizophrenia (*n* = 4; 3%), and other disorders (*n* = 7; 5%). The index offenses, i.e., the respective offense that led to the current admission, were as follows: 38 (29%) patients were convicted because of violations of the Narcotics Act; 28 (22%), because of aggravated assault; 19 (15%), because of rape or sexual assault; 15 (12%), because of homicide; 11 (8%), because of robbery; 9 (7%), because of theft; 4 (3%), because of arson; and 6 (5%), because of other offenses. A total of 26 (20%) patients had no educational qualifications; 59 (45%) had completed school to the end of grade 9 (“Hauptschulabschluss”), 32 (25%) had completed school to the end of grade 10 (“Realschulabschluss”), and 13 (10%) had graduated high school (“Abitur”).

### Procedures

The study was funded by the Ministry of Social Affairs, Free State of Bavaria, Germany, and approved by the ethics committee of the University of Ulm, Germany (application number: 174/17). It was performed in accordance with the Declaration of Helsinki.

Patients were informed about the study objectives and about the fact that neither participation nor non-participation would have any advantages or disadvantages with respect to their treatment. After receiving this information, they could decide whether they were willing to participate in the study or not. Patients who agreed to participate gave written informed consent and received a sheet with contact details. Participants were able to withdraw their consent at any time. The study protocol included instructions on how to inform the patient and therapist if the assessments indicated an acute risk of self-harm. Patients received neither financial nor non-financial compensation for their participation. They completed the questionnaires in small groups in a separate room on the ward, and a research assistant was available to provide help.

### Assessments

In addition to collecting sociodemographic, clinical, and legal data (sex, age, education, duration of detention, diagnosis according to ICD-10, and index offense), we asked patients to complete three questionnaires measuring institutional restraint, psychological symptoms, and suicidal ideation.

#### Perceived Institutional Restraint

Perceived institutional restraint was measured with a translated and adapted version of the questionnaire Measuring the Quality of Prison Life (MQPL) (9). The questionnaire had been adapted to assess the specific living conditions in forensic inpatient treatment and perceived therapeutic support (10). The aMQPL consists of 64 items assigned to the following 11 subscales: *Entry to forensic psychiatry* (4 items, Cronbach’s alpha = .599), *Relationship with fellow inmates* (4 items, Cronbach’s alpha = .678), *Relationship with caregivers* (4 items, Cronbach’s alpha = .843), *Relationship with therapists* (7 items, Cronbach’s alpha = .860), *Family contact* (3 items, Cronbach’s alpha = .588), *Transparency of procedures and decisions* (7 items, Cronbach’s alpha = .810), *Fairness* (5 items, Cronbach’s alpha = .817), *Respect* (6 items, Cronbach’s alpha = .827), *Safety* (6 items, Cronbach’s alpha = .800), *Quality of accommodation* (11 items, Cronbach’s alpha = .788), and *Therapeutic offerings/personal development* (7 items, Cronbach’s alpha = .853). The items were answered on a five-point Likert scale ranging from 1 (= I agree completely) to 5 (= I completely disagree). To evaluate the scores, we calculated the mean value of the subscales and of the entire scale. The higher the mean value, the more positively patients assess individual aspects of their quality of life (= subscales) or their overall quality of life (= total score). The aMQPL questionnaire has proven good reliability (Cronbach’s alpha of total score: *r* = 0.951). The factor structure was analyzed by confirmatory factor analysis [χ²(1,897) = 3,442.143; *p* < .001; Bollen–Stine bootstrap corrected *p* value = .008; Root mean square error of approximation (RMSEA) = .067; 90% confidence interval: .064–.071]. To assess perceived institutional coercion, we performed additional analyses that included the total score and focused on the following three subscales: *Transparency of procedures and decisions* (example item “When important decisions are made about me, I am told how they came about”), *Fairness* (example item “Staff here treat patients fairly when applying the rules”), and *Respect* (example item “I feel cared about most of the time in this hospital”).

#### Assessment of Psychological State

The BSCL (11) is a self-assessment instrument for measuring a broad range of psychological problems; the original version was published by Derogatis and Melisaratos in 1983 (12). The checklist contains a total of 53 items (total scale Cronbach’s alpha = .97), distributed over the following nine subscales: *Hostility* (5 items, Cronbach’s alpha = .72), *Anxiety* (6 items, Cronbach’s alpha = .80), *Depression* (6 items, Cronbach’s alpha = .88), *Paranoid ideation* (5 items, Cronbach’s alpha = .80), *Phobic anxiety* (5 items, Cronbach’s alpha = .72), *Psychoticism* (5 items, Cronbach’s alpha = .81), *Somatization* (7 items, Cronbach’s alpha = .80), *Interpersonal sensitivity* (4 items, Cronbach’s alpha = .80), and *Obsession–compulsion* (6 items, Cronbach’s alpha = .85). An example of one item on the *Hostility* subscale is “How much are you bothered by feeling easily annoyed or irritated?” The items are answered on a five-point Likert scale from 0 (“not at all”) to 4 (“extremely”). For the evaluation, we calculated the arithmetic mean of the subscale items and the total scale (global score). According to the authors of the German version, the test–retest reliability of the global score after 1 week was *r* = .87. The subscale *Depression* showed satisfactory convergent validity (*r* = .73) with Beck’s Depression Inventory (13) and various other clinical questionnaires (*r* = .36 to *r* = .83) (11).

#### Assessment of Suicidal Ideation

We used the German version of Beck’s Hopelessness Scale (BHS) (14) to assess suicidal ideation. The BHS contains 20 items, each of which can be answered with “true” or “false” (example item: “My future looks gloomy”). Values are summed to create a total score (maximum: 20 points). The authors of the scale assume that total scores >9 indicate an increased risk of suicide. According to the Kuder–Richardson Formula 20, reliability coefficients range from *r* = .72 to *r* = .97. The BHS discriminates well between people with and without suicidal ideation (Hedge’s *g* = .62 to 3.43) and also appropriately assesses the severity of suicidal ideation (Hedge’s *g* = 1.19 to 1.97).

### Data Analysis

A total of 12 linear regression analyses were calculated to assess the relationship between perceived institutional coercion and psychological state. The dependent variable was the BSCL global score or the subscale scores for *Hostility* and *Depression*. Predictors were the aMQPL total score or the mean values of the subscales *Transparency of procedures and decisions*, *Fairness*, and *Respect*, as well as sex and age.

In a next step, we used a binary logistic regression model to examine whether suicidal ideation (defined as total scores >9 in the BHS) was statistically predicted by the aMQPL total score; the mean values of the subscales *Transparency of procedures and decisions*, *Fairness*, and *Respect*; sex; and age.

To check whether patients’ perceptions might be associated with the diagnosed mental disorder, we conducted an additional five linear regression analyses to statistically predict the aMQPL total score on the basis of the variables *duration of detention* (in months), *substance-related disorder* (yes/no), *personality disorder* (yes/no), *schizophrenia* (yes/no), and *depression* (yes/no). Sex and age were included as additional predictors.

As outlined in Section 2.1, all analyses were based only on cases without missing values (number of missing values by questionnaire: BSCL *Hostility*, *Depression*, and *Global* score *n* = 3; BHS *n* = 18; aMQPL *n* = 53). A complete case analysis implicitly assumes that a missing value is not related to the respective outcome. Thus, we assumed that participants with missing values did not systematically differ from those without missing values. The validity check showed no systematic violations of this assumption: BSCL *Hostility* *t*(179) = −.350, *p* = .726; BSCL *Depression* *t*(179) = −.735, *p* = .463; BSCL total score *t*(179) = .047, *p* = .963; BHS χ²(1) = 2.662; *p* = .137.

Data were analyzed with IBM SPSS Statistics for Windows Version 25 (Armonk, NY: IBM Corp).

## Results

### Perceived Restraint and Psychological Symptoms

The results of the linear regression models to examine statistical predictors of psychological distress are shown in **Table 1** for the BSCL subscales *Hostility* and *Depression* and the *Global* score. The aMQPL total score was a significant predictor of the BSCL *Hostility*, *Depression*, and *Global* scores. Patients who rated their institution positively in terms of restraint had lower scores on the subscales *Hostility* (  *f*  ² = .27; medium effect size) and *Depression* (  *f*  ² = .15; small effect size) and had a lower *Global* score (  *f*  ² = .20; medium effect size) (15). A similar result emerged for the subscales *Transparency of procedures and decisions* and *Respect*. The more positive the patients’ rating for institutional transparency and respect, the less psychological distress they experienced. This applied to both the *Global* score and the two subscales *Hostility* and *Depression*. The aMQPL subscale *Fairness*, on the other hand, was only related to the BSCL subscale *Hostility*; i.e., the higher the perceived level of *Fairness*, the lower the *Hostility* score. Furthermore, the *Hostility* score was influenced by age; i.e., younger patients had higher levels of *Hostility*. The *Global* score was influenced by sex; i.e., female patients had higher global scores than male patients.

**Table 1:** Results of four linear regression models predicting the Brief Symptom Checklist score for the subscales *Hostility* and *Depression* and the *Global* score.

	Brief Symptom Checklist								
	Hostility			Depression			Global score		
	b	SE(*b*)	beta	b	SE(*b*)	beta	b	SE(*b*)	beta
Sex (reference category: male)	.276	.143	.154	.220	.156	.118	.292*	.122	.196*
Age (in years)	−.009*	.005	−.165*	−.007	.005	−.113	−.005	.004	−.106
aMQPL Total score	−.502**	.108	−.376**	−.432**	.118	−.312**	−.373**	.093	−.336**
*R* ^2^	.21			.13			.17		
aMQPL *Transparency* ^1^	−.364**	.069	−.416**	−.282**	.076	−.311**	−.260**	.059	−.357**
aMQPL *Fairness* ^1^	−.135*	.063	−.193*	−.081	.067	−.112	−.074	.053	−.127
aMQPL *Respect* ^1^	−.353**	.071	−.399**	−.323**	.077	−.352**	−.261**	.061	−.354**

b, unstandardized regression coefficient; SE(b), standard errors; beta, standardized regression coefficient; **p < .001; *p < .05; R^2^, proportion of the variance in the dependent variable that is predictable from the independent variables; ^1^Predictors sex and age were included in the model but are not displayed in the table.

### Perceived Restraint and Suicidal Ideation

The results of the binary logistic regression to examine which variables statistically predicted the likelihood of suicidal ideation, i.e., a BHS score > 9, are shown in **Table 2**. Perceived institutional restraint was a significant predictor, and patients who experienced little institutional restraint were less likely to have suicidal thoughts. Specifically, each additional point on the aMQPL total score reduced the risk of exceeding the BHS cutoff score by 4 (OR = 4.385; large effect size) (16). This relationship was also found for the subscales *Transparency of procedures and decisions* and *Respect*.

**Table 2:** Results of four binary logistic regression models predicting suicidal ideation of Beck’s Hopelessness Scale.

	Beck’s Hopelessness Scale			
	b	SE(*b*)	Exp(*b*)	95% Confidence interval Exp(*b*)
Sex (reference category: male)	−.185	.631	.831	.241–2.864
Age (in years)	.007	.022	1.007	.965–1.051
aMQPL Total score	1.478*	.520	4.385*	1.584–12.141
Nagelkerke’s *R* ^2^		.115		
aMQPL *Transparency* ^1^	.894*	.330	2.445*	1.280–4.672
aMQPL *Fairness* ^1^	−.042	.252	.959	.585–1.570
aMQPL *Respect* ^1^	1.190*	.346	3.289*	1.669–6.481

The binary dependent variable is being above the cutoff score (reference category: > 9); b, unstandardized regression coefficient; SE(b), standard error; Exp(b), odds ratio; **p < .001; *p < .05; ^1^Predictors sex and age were included in the model but are not displayed in the table.

### Perceived Restraint and Duration of Detention/Diagnosis

The results of linear regression models statistically predicting the aMQPL total score showed that neither the diagnosed mental disorder nor the duration of detention was associated with perceived institutional restraint (see **Table 3**).

**Table 3:** Results of five linear regression analyses predicting the adapted version of the Measuring the Quality of Prison Life (aMQPL) total score.

	b	SE(*b*)	beta	*R*²
Duration of detention	.001	.001	.135	.055
Substance-related disorder	−.069	.090	−.068	.045
Personality disorder	−.025	.098	−.023	.041
Schizophrenia	.197	.109	.158	.066
Depression	−.038	.136	−.025	.042

b, unstandardized regression coefficient; SE(b), standard errors; beta, standardized regression coefficient; **p < .001; *p < .05; R^2^, proportion of the variance in the dependent variable that is predictable from the independent variables. In all analyses, sex and age were included as predictors but are not displayed in the table.

## Discussion

The present study aimed to examine the association between perceived institutional restraint and forensic psychiatric inpatients’ psychological state. To our knowledge, the data are among the first to describe the relationship of psychopathological symptoms and perceived restraint in mentally disordered offenders.

The main result of our study was that the assessed aspects of institutional coercion (aMQLP total score and *Transparency of Procedures and Decisions* and *Respect* subscale scores) correlated with distinct psychological symptoms, namely, hostility and depression, whereas the* Fairness* subscale was only associated with hostility. Furthermore, the aMQLP total score was a significant statistical predictor of these symptoms. However, because of methodological limitations, causal conclusions cannot be drawn, and our results must be interpreted with caution. Nevertheless, our study might be able to add the aspect of psychopathology to previous findings of an association between perceived restrictiveness and both a caring vs. custodial focus and the individual risk assessment (8). One interpretation of the results might be that certain psychopathological symptoms are a result of dysfunctional adjustment to restrictive external conditions and that specific institutional characteristics might provoke specific symptoms (illustrated by the sole association of the *Fairness* subscale with hostility). In line with this interpretation, perceived restraint of forensic–psychiatric inpatients could have similar negative effects as physical coercion (fear, loss of dignity, humiliation, and fearful loss of control) (7). This interpretation would correspond with the concept of self-efficacy, defined as the personal judgment of “how well one can execute courses of action required to deal with prospective situations” (p. 122) (17). One can hypothesize that a subgroup of forensic psychiatric inpatients experience their personal situation as being more out of internal control; this group would then have to be considered as especially vulnerable.

On the other hand, our findings might also be interpreted as the result of the patients’ original mental disorder or personality traits (e.g., neuroticism) that were not assessed by our study design. However, as further statistical analysis showed, the diagnosis leading to admission had no influence on the perceived restraint. Nevertheless, co-occurring disorders or symptoms could have developed during detention, and they were not considered.

Furthermore, in our study, younger patients had higher hostility scores on the BSCL and female patients had a higher global symptom score. This result has to be interpreted in the context of the previous finding that in forensic psychiatry, younger patients tend to be secluded more often than older patients (2). If subgroups of patients actually are exposed to restrictive measures more often than others, they will probably perceive the institution as more restrictive. Both observations might also indicate different vulnerability levels—or different reporting behaviors—in subgroups of patients.

Another important factor that has to be considered is the duration of detention. The mean length of stay in our sample was nearly 3 years. According to previous studies, individuals in long-term detention experience a high amount of qualitative and quantitative symptom burden (18, 19). Although we did not find an association between duration of detention and perceived restraint, we cannot rule out that the psychological state is associated with the time spent in detention rather than with perceived restraint.

One of the major limitations of this study is the cross-sectional design, which does not allow causal conclusions to be drawn. The terms *predict* and *predictor* are used in conjunction with regression analysis and should be understood in a statistical sense only. It might also be the case that certain psychopathological symptoms lead to institutional conditions being perceived as restrictive, which would also be a possible explanation for each of the assessed symptoms (depression, hostility, and suicidal ideation). A further limitation is that we used only self-rating instruments to assess psychological symptoms and suicidal ideation. Combining self- and observer ratings and collecting data about suicide attempts and other self-harm from the medical records would probably have contributed to obtaining more robust data on the participants’ psychopathological state. Additionally, we did not classify the participants’ current psychological state according to common diagnostic criteria (DSM, ICD); thus, we were not able to exclude participants with disorders that had developed during detention. Such disorders (e.g., depressive episodes) might also have influenced the self-rating scales. Additionally, we did not use the established scales of the MQLP but an adapted version, the aMQLP; however, preliminary statistical analysis showed sufficient validity of the aMQLP. Finally, our results are from the German forensic mental health care system and are not generalizable to other countries. In our opinion, the participating hospitals properly represent low- and medium-security forensic mental health institutions in Germany; however, there may have been a selection bias in that only patients who felt overly restricted may have participated in the study.

In conclusion, our results might contribute to the research on institutional coercion in forensic psychiatry, because they add the aspect of individual psychopathology to the concept of perceived restriction in forensic–psychiatric care. Forensic mental health care professionals should be aware of perceived restraint as a potential indicator for the development of distinctive psychopathological symptoms and vice versa. Further research might use a design that allows causal conclusions to be drawn and also include a larger sample of inpatients and institutions. Additionally, the subjective perception of institutional restraint should be compared with an objective rating to control for individual factors (e.g., psychopathology, diagnosis, and personality). It would be of further interest to assess the influence of perceived institutional coercion on the therapeutic relationship and on specific outcome measures, such as disorder-specific psychopathology or the risk of re-offending.

## Ethics Statement

All patients were informed about the objectives of the study, and all provided written informed consent. The study protocol included instructions on how to inform the patient and therapist if the assessments indicated an acute risk of self-harm. Patients received neither financial nor non-financial compensation for their participation. They completed the questionnaires in small groups in a separate room on the ward, and a research assistant was available to provide help. The study was approved by the local ethics committee (Ulm University, Ulm, Germany, No. 176/17).

## Author Contributions

MD, JS, IF, and MB designed the study; MB collected the data; JS analyzed the data; and JS, MD, and IF interpreted the data. IF wrote the initial draft of the manuscript. All authors had full access to all the data in the study and take responsibility for the integrity and accuracy of the data analysis. All authors contributed to, read, and approved the final version of the manuscript.

## Funding

Data published in this paper are part of a research project of the Department of Forensic Psychiatry and Psychotherapy of Ulm University (head: Prof. M. Dudeck), funded by the Ministry of Social Affairs, Free State of Bavaria, Germany.

## Conflict of Interest Statement

Data published in this paper are part of a research project of the Department of Forensic Psychiatry and Psychotherapy of Ulm University (head: MD), funded by the Ministry of Social Affairs, Free State of Bavaria, Germany. The remaining authors declare that the research was conducted in the absence of any commercial or financial relationships that could be construed as a potential conflict of interest.
